# Cooking Skills, Eating Habits and Nutrition Knowledge among Italian Adolescents during COVID-19 Pandemic: Sub-Analysis from the Online Survey COALESCENT (Change amOng ItAlian adoLESCENTs)

**DOI:** 10.3390/nu15194143

**Published:** 2023-09-25

**Authors:** Silvia Marconi, Loredana Covolo, Monica Marullo, Barbara Zanini, Gaia Claudia Viviana Viola, Umberto Gelatti, Roberto Maroldi, Nicola Latronico, Maurizio Castellano

**Affiliations:** 1 Department of Clinical and Experimental Sciences, University of Brescia, 25123 Brescia, Italy; monica.marullo@unibs.it (M.M.); barbara.zanini@unibs.it (B.Z.); gaia.viola@unibs.it (G.C.V.V.); maurizio.castellano@unibs.it (M.C.); 2 Department of Medical and Surgical Specialties, Radiological Sciences and Public Health, University of Brescia, 25123 Brescia, Italy; loredana.covolo@unibs.it (L.C.); umberto.gelatti@unibs.it (U.G.); roberto.maroldi@unibs.it (R.M.); nicola.latronico@unibs.it (N.L.)

**Keywords:** cooking skills, adolescent, healthy eating, ultra-processed food, nutrition knowledge, food skills, lifestyle medicine

## Abstract

Background: Cooking skills (CS) have the potential to improve self-care behaviours and healthy development among adolescents. The COVID-19 pandemic has affected lifestyles worldwide, and the present study aims to investigate the level of CS after the pandemic, as well as its associations with nutrition knowledge and eating behaviours among a cohort of Italian adolescents. Methods: We submitted an online survey about lifestyle changes to students aged 13–21 years during the pandemic. Based on overall culinary abilities, we divided respondents into high, medium and low CS. Worsening or improvement in diet quality was detected by assigning an eating habit index (EHI; 0–54). Results: Out of the 1686 questionnaires collected, 21.5%, 63.6% and 14.9% reported high, medium and low CS, respectively. The EHI scores were statistically higher among students who were able to cook more than 20 recipes compared to those reporting no cooking abilities (30.2 ± 5.9 vs. 26.6 ± 5.7; *p* = 0.0001). High CS significantly correlated with better EHI (OR 1.44; *p* = 0.009), lower consumption of ultra-processed food (OR 1.80; *p* < 0.0001) and better nutrition knowledge (OR 1.42; *p* = 0.007). Conclusions: Adolescents with good CS showed better nutrition knowledge and healthier eating habits, including lower consumption of ultra-processed foods. Projects aimed to improve CS in adolescents can therefore promote healthier development.

## 1. Introduction

A growing body of evidence suggests that knowing how to prepare and cook food is a valuable life skill that is associated with several health benefits, including better diet quality, increased recognition and consumption of healthier foods, weight control, and even longevity [1,2,3]. Moreover, a good level of cooking skills (CS) has been associated with higher vegetable, fruit and micronutrient intake [4] while the lack of culinary knowledge can constitute a barrier to the preparation of healthy meals and promote an increased consumption of fast and ultra-processed foods [1,5].

Adolescence, a life stage clearly marked by biological, psychological and emotional changes, is a key period to develop and reinforce eating behaviours that are likely to influence future health [6]. The involvement of adolescents in food preparation has been related to better diet quality and better eating patterns throughout life [7,8]. Recent studies have reported that learning CS earlier in life, i.e., during infancy or adolescence, positively affects the development of cooking abilities during adulthood and produces a positive long-term effect on health [1]. Lavelle et al. reported that people who learned CS during childhood or adolescence, rather than during adulthood, were more likely to taste new foods, showed better food safety knowledge, and consumed fewer savoury foods and industrial pastries [9].

Most human behaviours are learned through observation and modelling, and mothers are mentioned as the primary source in developing CS among their daughters and sons [1,2]. The evolution of society and the changing role of women, who are increasingly engaged with work outside the home, has dramatically reduced the time available for both cooking and transferring CS to their children. This could imply a consequent lack of opportunities for young people to learn basic CS [9,10]. On the other hand, digitalization and the availability of cooking tutorials and online cooking courses seem to compensate for this lack and can represent an alternative to improve CS during childhood and adolescence.

Lockdown restrictions enforced during the COVID-19 pandemic have profoundly affected lifestyles worldwide, especially among adolescents. The closure of schools, the suspension of outdoor sports and activities, physical distancing, and isolation have had a strong impact [11,12,13]. Many studies have also reported changes in eating behaviours; the shift from eating outside to mandatory in-house food consumption, together with changes in food availability and accessibility, has altered the dietary profiles of many people [14,15]. In addition, changes in family cohabitation and work dynamics, such as the introduction of remote working, have influenced the preparation and sharing of meals within the home [16].

The aim of the present study was to assess CS levels and to identify any correlation between eating habits and nutritional knowledge two years after the COVID-19 pandemic in a sample of Italian adolescents. As part of an epidemiological study designed to assess long-term changes in several aspects of lifestyle, COALESCENT [17,18], we performed an online survey among students attending high schools in the Brescia district. This is a province in Northern Italy that was one of the Italian areas most affected by the spread of COVID-19 and the resulting restrictions. The results of this sub-analysis on CS represent a starting point for future interventions aimed at promoting healthy nutrition and well-being in this target population.

## 2. Materials and Methods

### 2.1. Survey Methodology

The COALESCENT (Change amOng ItAlian adoLESCENTs) survey was conducted in collaboration with the Territorial School Office of the Brescia district. Among the 57 high schools, both public (state-funded) and private, to which the project was presented, eight schools joined the survey. Among these schools, students willing to voluntarily participate in the survey provided informed consent, which had to be signed by the participants’ parents or guardians in the case of underage students, or by students themselves if over 18. Participants independently completed the online survey during school hours in a supervised classroom setting. The survey, developed with the Lime Survey software v5.2.4, included an introductory page describing the aim of the project and information on the ethics, and each questionnaire was sent to a final database and downloaded as a Microsoft Excel sheet [17,18]. Students were assured that all their responses would be used only for the aims of the study and would be treated according to the criteria of anonymity and confidentiality, and they were able to withdraw their participation in the survey at any stage before submission. Incomplete questionnaires were not saved.

### 2.2. Questionnaire

The structured survey included 110 questions, divided into eight sections (A–H; [17,18]): (A) consensus and age (2 questions); (B) personal data (including 2 questions about gender and school attended and 2 questions reported weight in kg and height in cm); (C) housing information (including 4 questions about family composition, cohabitation situation, availability of gardens and/or balconies); (D) educational level and professional situation of parents or caregivers before and during the pandemic (6 questions); (E) eating behaviours (56 questions were presented to be answered regarding “before” and “during” confinement conditions); (F) other lifestyle aspects, including 26 questions modified from validated tools [18]: exploring physical activity, sleeping habits, smoking, smart phone and digital devices addiction; (G) emotional and psychological aspects (including 6 questions from validated tools [19,20,21] to assess the emotional aspects such as stress, anxiety, depressed mood and level of concern during confinement condition); (H) nutritional knowledge (including 6 questions from the validated Moynihan questionnaire) [22].

The section on eating behaviours aimed to assess whether the intake of selected foods (fruit and vegetables, cereals and starchy foods, sweets, protein-based foods, beverages and condiments, and ultra-processed foods) was increased, reduced or unchanged during the two years of COVID-19 restrictions in Italy (modified from Di Renzo et al.) [23]. In addition, questions about food availability, who supervised the cooking process, and appetite, as well as questions on comfort food consumption and weight changes, were included. CS was assessed through two specific questions. Cooking abilities were assessed through the question, “How many dishes are you able to cook by yourself? (Do not consider ready-made dishes that only need to be defrosted or reheated)”. Response options included: None; From 1 to 9; From 10 to 19; More than 20. The second question evaluated changes in CS during the pandemic: “Since the beginning of the pandemic, have you learnt to cook anything?” with 4 options: No; Yes, I have acquired some skills in cooking; Yes, I have acquired many skills in cooking; I don’t know. Based on the given answers, the population was divided into three groups: high CS (≥ 10 self-cooked dishes and many CS acquired during pandemic); low CS (No CS or no CS acquired during pandemic); medium CS (all other combinations, including also ‘I don’t know’).

Based on changes in the consumption of several foods, we assigned an eating habit index (EHI) score from 0 to 54, reflecting a worsening or an improvement in diet quality, respectively, compared to the period before the pandemic [17,18].

Regarding nutrition knowledge, the population was divided based on the number of correct answers: “good nutrition knowledge” (≥4 correct answers); “limited nutrition knowledge” (0 to 3 correct answers).

Based on WHO guidelines on physical activity and sedentary behaviours, we referred to adolescents who met WHO requirements as “active” [24].

### 2.3. Ethical Considerations

To guarantee students’ confidentiality, their personal information and answers were anonymised in accordance with the provisions of the General Data Protection Regulation (GDPR 679/2016). Due to the anonymous nature of this survey, personal data could not be traced and, consequently, did not require the approval of the local ethics committee. The online survey was conducted in full agreement with national and international regulations in compliance with the Declaration of Helsinki 2000 [17,18].

### 2.4. Statistical Analysis

The analyses included descriptive statistics (i.e., frequencies and percentages for categorical variables and mean values with standard deviations for continuous variables). Comparisons between groups were made using the χ^2^ test or Fisher’s exact probability test for categorical variables and the Mann–Whitney test for continuous variables. A binary logistic regression model was carried out, with CS as the dependent variable. The covariates to be included in the final model were selected on the basis of univariate analysis, with a univariate *p* value < 0.05 as the main criterion. Then, using a backward selection process, statistically non-significant variables were excluded. To check for collinearity among the variables, the Spearman correlation test was used. The results of the logistic regression are reported with adjusted odds ratios and 95% confidence intervals. A *p*-value less than 0.05 was considered statistically significant for all analyses. Statistical analyses were performed using STATA (Stata Statistical Software: Release 16.0 College Station, TX, USA: Stata Corporation).

## 3. Results

### 3.1. Descriptive Analysis

**Figure 1:**
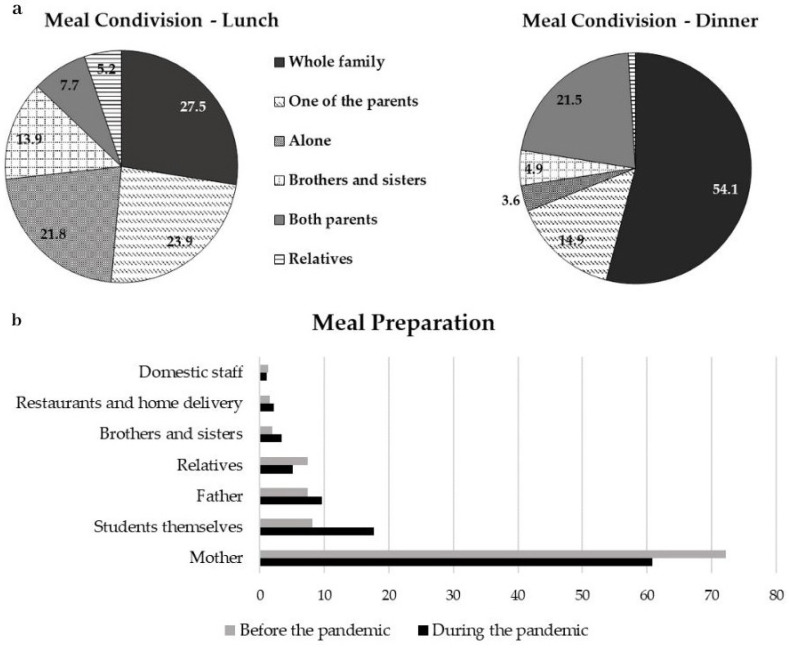
Meal condivision and management: (**a**) The evaluation of meal management showed that since the beginning of the pandemic, the students had lunch must of the time with the whole family (27.5%), with a parent or guardian (23.9%), or alone (21.8%). As for dinner, most of the students ate with the whole family (54.1%), with both (21.5%) or with one of parents (14.9%). (**b**) Changes in meal preparation. Before the pandemic, in 72.2% of the cases, mothers prepared the meals and 8.1% the students themselves. After the pandemic, the number of mothers involved in meal preparation was reduced to 60.6%, while the number of adolescents who cooked themselves more than doubled (17.3%).

**Figure 2:**
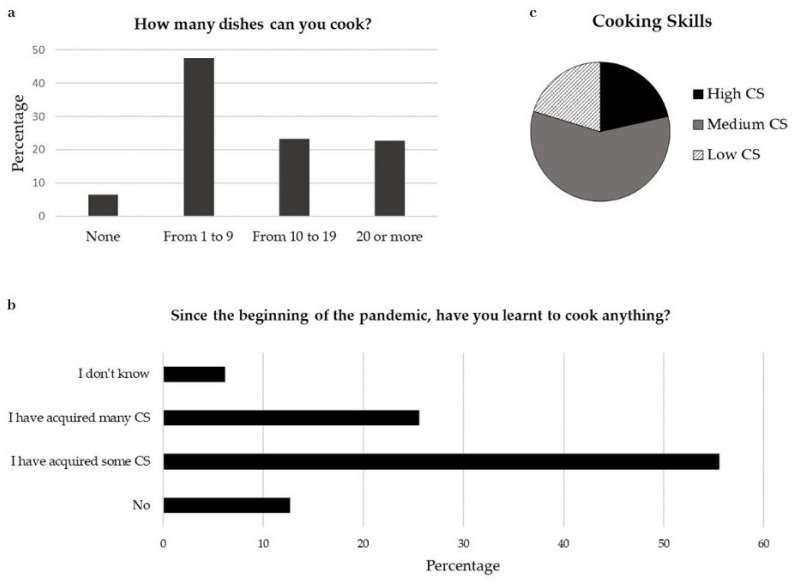
Cooking skills description: (**a**) The percentage of students who stated that they could cook from 1 to 9 dishes is 47.6%, from 10 to 19 dishes 23.3% and 20 or more dishes is 22.6%; (**b**) Since the beginning of the pandemic, 55.6% of the subjects stated that they had acquired some cooking skills, 25.6% that they had acquired several cooking skills, while the remaining population reported no cooking skills acquisition (12.7%) or they answered “I don’t know” (6.2%); (**c**) According to these answers population was divided into High CS (21.5%), Medium CS (63.6%) and Low CS (14.9%).

- Sample description. The online survey was completed by 1686 students (response rate 34.6%) attending eight different high schools within the Brescia district; half of the schools involved were public (state-funded) and half private, totalling 4866 students. The study population comprised students aged 13 to 21 years old and was administered between December 2021 and February 2022. Of the respondents, 50.2% were females, and 46.6% were males; 3.2% of the sample chose not to declare their gender. The average age of the sample was 15.8 ± 1.6 years, ranging from 13 to 21 years. Regarding the Body Mass Index (BMI = kg/m^2^, calculated from self-reported height and weight values), females registered an average value of 20.6 ± 3.0 (ranging from 13.2 to 36.1), males an average value of 21.7 ± 3.8 (ranging from 14.6 to 55.6) and the group that did not declare gender an average value of 22.6 (ranging from 12.5 to 50.2). Since the beginning of the pandemic, 37.5% of the population reported an increase in weight and 27.3% a reduction in weight, while 8.4% declared no change. 21.5% of the study population answered “I don’t know” about the weight change and 5.2% preferred not to answer.
- Meal management. The evaluation of meal management showed that during the past year, students had lunch must of the time with their whole family (27.5%), with a parent or guardian (23.9%), or alone (21.8%). As for dinner, most of the students ate with their whole family (54.1%), with both (21.5%) or one of their parents (14.9%), and 3.6% had dinner alone (Figure 1a). The study also revealed changes in meal preparation; before the pandemic, in 72.2% of the cases, meals were prepared by mothers and in 8.1% of cases by the students themselves. After the pandemic, the number of mothers involved in meal preparation decreased to 60.9%, while the number of adolescents who cooked by themselves more than doubled (17.7%) (Figure 1b).
- Eating Habits (Eating Habit Index, Ultra-processed food, and comfort food consumption). EHI scores ranged from 12 to 49, with a median score of 28 and a mean value of 28.6 (±6.0 SD).
  We analysed ultra-processed food intake, specifically investigating the frequency of consumption of industrially packaged foods, such as biscuits, snacks, chips, sweets, soft drinks, frozen pizza or ready-to-eat meals. Based on the frequency of consumption, we divided the population into high (daily), intermediate (weekly) and low/never (monthly or never) consumption. Compared to the pre-pandemic period, the rate of adolescents reporting high consumption of ultra-processed foods increased from 45.7% to 48.1%, while intermediate consumption was reduced from 28.7% to 25.5%. We also observed a slight increase in the percentage of adolescents reporting low consumption of ultra-processed foods (from 25.7% to 26.4%); none of these trends were statistically significant.
  When asked if food was ever consumed to seek consolation, 15.7% of the students answered “yes, for both the period before and during the pandemic” and 16.5% reported an increase in consumption compared to the pre-pandemic period; 5.5% reported a reduction in comfort food consumption. The remaining population did not consume food for consolation (49.0%) or did not know how to answer (13.4%).
- Cooking skills. Most of the adolescents involved in the present study stated they were able to cook from 1 to 9 dishes (47.6%), 23.3% were able to prepare from 10 to 19 dishes and 22.6% declared they could cook more than 20 different dishes. A total of 6.5% reported that they were not able to cook a meal (Figure 2a). Since the beginning of the pandemic, 55.6% of the subjects stated that they had acquired some cooking skills, 25.6% that they had acquired several cooking skills, while the remaining population reported no cooking skill acquisition (12.7%) or they answered “I don’t know” (6.2%) (Figure 2b). As previously described, based on these answers, the population was divided into 3 different levels of culinary competence: high CS (21.5%), medium CS (63.7%) and low CS (14.9%) (Figure 2c).
- Nutritional knowledge. The assessment of nutritional knowledge showed that 70.5% of the population correctly answered 1 to 3 out of 6 questions, thus showing limited knowledge, while 29.5% of the students showed good nutrition knowledge by correctly answering 4 or more questions.

### 3.2. Correlation of Eating Habit Index with Considered Variables

**Figure 3:**
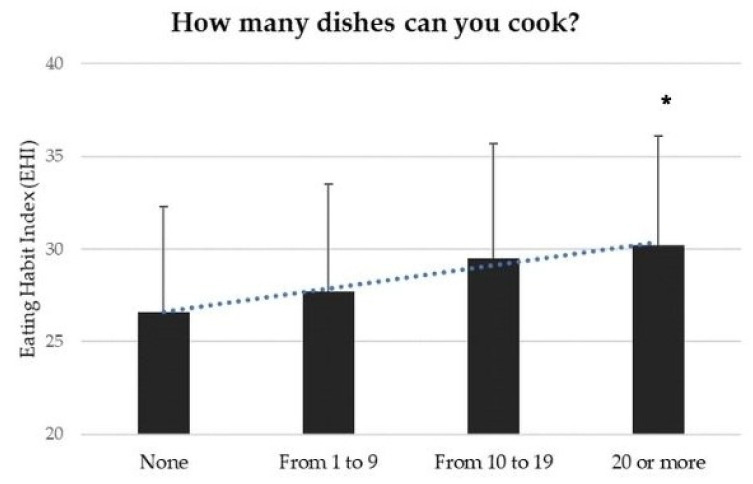
Correlation between EHI and cooking skills. Adolescents who were able to cook more than 20 recipes had an EHI of 30.2 ± 5.9, which was significantly higher than those who had no CS, who reached an EHI of 26.6 ± 5.7 (* *p* = 0.0001).

The correlation between the EHI and CS showed a linear and progressive increase in the score within the four categories assessing the first question about CS; adolescents who were able to cook more than 20 recipes had an EHI of 30.2 ± 5.9, which was significantly higher than those who had no CS, who reached an EHI of 26.6 ± 5.7 (*p* = 0.0001) (Figure 3). According to a multivariate logistic analysis, an EHI score > 33 (75° percentile value) was associated with high CS (OR 1.61; *p* > 0.0001), good nutrition knowledge (OR 1.49; *p* = 0.002), low and moderate ultra-processed foods consumption during the pandemic (OR 2.20; *p* < 0.0001 and OR 1.46; *p* = 0.01, respectively), reduction of weight (OR 2.24; *p* = 0.001) and female gender (OR 1.33; *p* < 0.0001). The other variables did not show a significant correlation [17].

### 3.3. Correlation of Cooking Skills with Considered Variables

When analysing the characteristics of the population with high CS, a significantly positive correlation was observed in the case of female gender (OR 1.32; *p* = 0.041), students attending state schools (OR 1.97; *p* < 0.0001), with an overweight condition (OR 1.70; *p* = 0.02) and with a weight reduction during the pandemic (OR 2.10; *p* = 0.006). Moreover, the multivariate logistic regression significantly correlated higher CS with the ability to self-prepared meals both before and during the pandemic (OR 1.56; *p* = 0.031 and OR 2.99; *p* < 0.0001 respectively), with higher EHI scores (OR 1.44; *p* = 0.009), and moderate or lower ultra-processed food consumption (OR 1.46; *p* = 0.014 and OR 1.80; *p* < 0.0001 respectively). Finally, adolescents showing higher CS had significantly better nutrition knowledge compared to the remaining population (multivariate logistic analysis OR 1.42; *p* = 0.008) and were physically active (OR 1.32; *p* = 0.048) (Table 1).

The weak correlations among independent variables (ρ < 0.40) suggested that there was no collider that might affect the results, and therefore, no variables were removed.

## 4. Discussion

During the COVID-19 pandemic, a change in lifestyle occurred in all age groups and worldwide areas [11]. Italy, and especially the Brescia district, has been heavily affected both by the SARS-CoV-2 virus and isolation measures. Adolescents were among the population groups that were most affected by the restrictions in terms of school attendance, physical activity, leisure time and sociability [12]. To our knowledge, the COALESCENT study was the first project aiming to explore the long-term consequences of the lockdown period on several aspects of lifestyle, including cooking abilities and their possible correlation with eating habits and nutrition knowledge [17,18].

CS, referred to as the ability to perform tasks related to the planning and preparation of meals starting from unprocessed foods and culinary ingredients, is increasingly included in strategies to prevent and reduce obesity and the prevalence of diet-related chronic non-communicable diseases. It has been demonstrated in several studies that better levels of cooking skills are associated with an improvement in overall diet quality and an increase in the intake of micronutrients that can improve health [25,26,27]. Moreover, a recent prospective cohort study showed that being actively involved in preparing one’s own food was positively associated with a reduction in the risk of obesity [28].

It has been observed that the period of life during which CS is acquired influences future eating habits: learning CS during childhood or adolescence is positively associated with subsequent use of CS and cooking attitude in adult life [9]. Since adolescence is a period of life in which future habits are shaped, assessing CS and eating habits among adolescents can be crucial for the design of health interventions, even more so after the recent pandemic [13].

To our knowledge, the present study was the first to investigate CS and eating habits among a population of Italian adolescents. The results indicate that CS significantly and directly correlates with better eating and higher diet quality, expressed by the EHI. In addition, adolescents with higher CS show significantly better nutrition knowledge, consumption of fewer ultra-processed foods and, as expected, higher autonomy in meal management.

One of the novel points of our study is that, among the difficulties and negative repercussions documented during the pandemic, time spent at home gave people the opportunity to acquire and/or improve CS. Our results showed that spending time cooking produced a marked improvement in eating habits in this adolescent population, particularly when affected by the restrictions enforced during the pandemic. These data, although not representative of the entire nation, underline once again the central role played by culinary knowledge in health promotion. The majority of our population declared to know how to cook “from scratch”, and only 6.5% were not able to cook any meal; additionally, more than the 55% and 25% of our sample reported having acquired “some” or “several” CS since the beginning of the pandemic. Overall, less than 15% of the adolescents involved in the study fell under the Low CS category, and this result is similar to what Neves and co-workers recently observed in the Brazilian adolescent population, where more than two-thirds reported knowing how to cook “from scratch” [29]. Similar rates were also observed a few years ago in a study among adolescents in New Zealand, where approximately 80% of students reported that they were able to cook a recipe using basic ingredients, and their ability was positively associated with better nutritional indicators [2]. In our population, we observed a linear improvement in diet quality and CS, expressed as the number of self-cooked dishes, underlying the robust relationship between cooking and health.

At the same time, the strong and significant correlation between high CS and lower consumption of ultra-processed foods found in our population is in line with what other researchers have observed; Lam and collaborators found an association between greater home-food preparation abilities and more frequent use of these skills and lower consumption of ultra-processed food [30]. High consumption of packaged ultra-processed food is strongly and significantly associated with weight gain and poor health outcomes [31]. Ultra-processed foods are ubiquitous, both in high-income and middle- and low-income countries, and home-cooking is recommended by public health authorities, as well as several food-based national dietary guidelines [26]. In a recent study by Brasington and collaborators, cooking confidence and creativity scale scores were low among consumers of ultra-processed ready-to-eat meals, suggesting that individuals lacking CS tend to consume higher amounts of ultra-processed foods, generally with lower nutritional value compared to meals prepared using raw ingredients [32].

Data on meal sharing indicates that dinner remains the main family gathering time and only a small percentage of students have dinner alone. These observations are in line with previous Italian surveys [33]. Family meal sharing appears to act as a protective factor for adolescents and may positively influence their health, creating opportunities to eat healthy foods and, for parents, to role-model healthy eating behaviours [2,34]. Of interest is that, during the pandemic, the number of students directly involved in meal preparation more than doubled. However, both before and during the pandemic, mothers were mainly responsible for meal preparation; the decline in percentage observed thus suggests that there has been a shift in some of the tasks from mothers to children. Currently, in Italy, the figure of the mother is primarily responsible for meal preparation, both before and after the pandemic [35]. The key role played by mothers, both in meal preparation and in passing on culinary skills, has been demonstrated in different studies. Most human behaviours are learned through observation and modelling, and mothers are mentioned as the primary source to develop CS [1]. The correlation between high CS and female gender observed in the present study emphasises women’s attitude toward healthy eating habits. A large body of literature has studied the relationship between gender and food preparation behaviours; women seem more likely than men to be involved in cooking, to spend time cooking and to feel confident in cooking [1,28,29]. UNESCO also defines women as custodians of the culinary tradition, with special reference to the Mediterranean Diet [33,35]. The evolution of society and the changing role of women, who are increasingly engaged with work outside the home, make it imperative to promote CS beyond possible gender stereotypes and to involve the entire population in both learning and applying the knowledge acquired [9]. To avoid this progressive decline of CS and knowledge, it is important to involve children and adolescents in meal planning, ingredient selection and recipe execution, also trying to pass on traditional local and family recipes.

The results of the present study confirmed a significant correlation between high CS and good nutritional knowledge and active lifestyle, as well as a significant correlation between better eating habits, detected by a high EHI. Considering that less than 30% of the adolescents involved showed a good level of nutritional knowledge, it would also be appropriate to promote projects aimed at fostering this topic. Similarly, it has been suggested that developing food and nutrition knowledge among adolescents may better equip individuals to achieve healthier dietary outcomes [22,36]. These results suggest that those who eat better (higher HEI), as well as have better nutrition knowledge, can manage meals independently, possess culinary skills and do not frequently consume packaged ready-to-eat meals.

A second positive aspect of novelty is represented by the significant correlation of higher CS not only with overweight but also with greater weight reduction during the pandemic. This finding leads us to hypothesise that individuals showing higher CS, despite being overweight, used their knowledge and skills during the pandemic to improve their dietary quality and thus managed to reduce their body weight. Indeed, students showing a higher EHI significantly correlate with weight reduction during the pandemic. Probably, the pandemic period, with a lot of time at home, was useful to implement CS, nutrition knowledge and overall diet quality, with positive effects on body weight [37].

Finally, in the multivariate logistic regression analysis, we found a significant correlation with high CS in the case of students attending state schools, and this result allows us to hypothesise that, as was also observed in a Brazilian study, CS are not the preserve of the economically advantaged classes but might allow for the inclusion of lower income statuses [29].

One of the weaknesses of the present study is the absence of extensive and validated measures of CS, but given the large number of questions, we decided not to increase them further. Moreover, self-referred CS could be considered a disadvantage of the present study. Another limitation is the localization of the survey in the Brescia district only, which is not representative of the whole country. Finally, the inability to access the data of students who voluntarily decided not to participate in the survey could represent a risk of bias. Our study has some strengths: the large cohort of adolescents involved is remarkable in our country and, for the first time in Italy, CS are investigated as part of a comprehensive lifestyle surveillance, giving us the possibility to look for correlations with all the other variables analysed.

The results of the COALESCENT study support the link between the use of unprocessed culinary foods and ingredients and healthier eating habits. In a social context where people are losing abilities and interest in home-cooking, projects that reverse this trend could promote the heath of present and future populations. At the same time, cooking is becoming a topic of entertainment, often far from the ideal of simple, healthy cooking; therefore, it would be important to draw attention to proposals that can be translated into healthy, everyday cooking. In recent years, the field of lifestyle medicine has seen widespread promotion of “teaching kitchens: learning laboratories where individuals and families learn nutrition information and practical skills that can promote well-being and manage chronic diseases [38]. Proposing similar “teaching kitchens” interventions focused on the adolescent population could be a valuable approach to counter the spread of suboptimal dietary patterns, which are associated with the majority of leading causes of death globally [39]. In addition, in recent studies, it has been shown how the use of innovative technological tools such as multidimensional games, videos and web-based tutorials can improve adolescents’ engagement and attention span [8]. Furthermore, proposing interventions involving a whole group of adolescents would allow the positive influence of peers to be harnessed [2].

## 5. Conclusions

Adolescence represents an ideal life stage to develop and reinforce healthy eating behaviours that will influence future health. Cooking is a valuable life skill that is linked to improved diet quality, increased recognition of healthier foods and reduced consumption of ultra-processed foods. Designing interventions that foster both nutritional knowledge and cooking skills in adolescents may be an important tool for improving and promoting healthier development.

## Figures and Tables

**Table 1:** Multivariate analysis of the significant association between CS and variables considered.

Multivariate Logistic Regression High Cooking Skills vs.:			
	OR	CI 95%	*p* Value
Gender (female)	1.32	1.01–1.73	0.041
BMI (overweight)	1.70	1.09–2.65	0.02
Type of school (state)	1.97	1.41–2.75	<0.0001
Weight during the pandemic (reduction)	2.10	1.24–3.55	0.006
Meal management (self-prepared meals before the pandemic)	1.56	1.04–2.35	0.031
Meal management (self-prepared meals during the pandemic)	2.99	2.23–4.00	<0.0001
Eating Habit Index (high score)	1.44	1.10–1.85	0.009
Ultra-processed food consumption during the pandemic (moderate)	1.46	1.08–1.97	0.014
Ultra-processed food consumption during the pandemic (low/never)	1.80	1.33–2.44	<0.0001
Nutrition knowledge (≥4 corrected answers)	1.42	1.10–1.85	0.007
Physical activity (active)	1.32	1.00–1.73	0.048

## Data Availability

The data presented in this study are available on request from the corresponding author.
